# MALL, a membrane-tetra-spanning proteolipid overexpressed in cancer, is present in membraneless nuclear biomolecular condensates

**DOI:** 10.1007/s00018-022-04270-w

**Published:** 2022-04-10

**Authors:** Armando Rubio-Ramos, Miguel Bernabé-Rubio, Leticia Labat-de-Hoz, Javier Casares-Arias, Leonor Kremer, Isabel Correas, Miguel A. Alonso

**Affiliations:** 1 Centro de Biología Molecular Severo Ochoa, Consejo Superior de Investigaciones Científicas, Universidad Autónoma de Madrid, 28049 Madrid, Spain; 2 Centro Nacional de Biotecnología, Consejo Superior de Investigaciones Científicas, 28049 Madrid, Spain; 3 Department of Molecular Biology, Universidad Autónoma de Madrid, 28049 Madrid, Spain

**Keywords:** Proteolipids, Biomolecular condensates, Membranes, PML bodies, Nuclear aberrations, Cancer

## Abstract

Proteolipids are proteins with unusual lipid-like properties. It has long been established that PLP and plasmolipin, which are two unrelated membrane-tetra-spanning myelin proteolipids, can be converted in vitro into a water-soluble form with a distinct conformation, raising the question of whether these, or other similar proteolipids, can adopt two different conformations in the cell to adapt their structure to distinct environments. Here, we show that MALL, another proteolipid with a membrane-tetra-spanning structure, distributes in membranes outside the nucleus and, within the nucleus, in membrane-less, liquid-like PML body biomolecular condensates. Detection of MALL in one or other environment was strictly dependent on the method of cell fixation used, suggesting that MALL adopts different conformations depending on its physical environment —lipidic or aqueous— in the cell. The acquisition of the condensate-compatible conformation requires PML expression. Excess MALL perturbed the distribution of the inner nuclear membrane proteins emerin and LAP2β, and that of the DNA-binding protein BAF, leading to the formation of aberrant nuclei. This effect, which is consistent with studies identifying overexpressed MALL as an unfavorable prognostic factor in cancer, could contribute to cell malignancy. Our study establishes a link between proteolipids, membranes and biomolecular condensates, with potential biomedical implications.

**Supplementary Information:**

The online version contains supplementary material available at 10.1007/s00018-022-04270-w.

## Introduction

Phase separation is a chemical–physical principle by which a well-mixed solution of components becomes de-mixed into two or more co-existing phases with uniform properties. It has been proposed that membrane-less compartments, for instance nuclear bodies (NBs), such as nucleoli, speckles, Cajal bodies and pro-myelocytic leukemia (PML) NBs, and other cellular assemblies, such as solid-like protein aggregates, heterochromatin and large signaling complexes, form by liquid–liquid phase separation induced by extensive multivalent protein–protein interactions [1–3]. This network of interactions drives the separation of a condense phase or “biomolecular condensate”, which contains the crosslinked proteins, and a light phase comprising all the other proteins [1]. Biological membranes also appear to be compartmentalized by phase separation. In this case, protein–protein, protein–lipid and lipid–lipid interactions within the membrane and formation of membrane–proximal biomolecular condensates likely contribute to membrane segregation into fluid regions and more condensed regions [4, 5]. The plasma membrane of myelin-forming cells, which is segregated into the myelin membrane and the membrane surrounding the cell body, provides an extreme example of a compartmentalized membrane [6]. Although, phase separation underlies the formation of both aqueous and membrane condensates, it is obvious that embedding in these two such distinct environments requires different protein structures.

Proteolipids were originally defined as proteins displaying unusual lipid-like properties that render them highly soluble in organic solvents used to extract cell lipids [7]. Several of the best characterized proteolipids are myelin proteins, such as the proteolipid protein (PLP) [8], plasmolipin (PLLP) [9] and MAL [10]. These three having a membrane-tetra-spanning structure, a distribution in condensed membranes [11–13], and preferentially partition into detergent-resistant membrane (DRM) fractions [14–16], which are enriched in proteins associated with condensed membranes [17]. An intriguing property of PLP [18, 19] and PLLP [9, 20], probably shared by other related proteolipids, is that they can be converted in vitro into a water-soluble form with a quite distinct conformation. This capacity long ago prompted the still unanswered question as to whether this type of proteolipid exists in the cell as one conformer or as two [21].

In addition to myelin-forming cells, the PLLP and MAL proteolipids are expressed by epithelial cells [9, 22–24] and, in the case of MAL, also by human T cells [25], and mediate distinct specialized routes of membrane trafficking [23–26]. The MALL-like (MALL) protein [27], also known as BENE [28, 29], together with PLLP, MAL and four other members, constitute the MAL-family of proteins [10, 30, 31]. MALL share with PLLP and MAL a membrane-tetra-spanning topology, high affinity for organic solvents, and preferential partitioning into DRM fractions [29]. In this study, using a new antibody to MALL, we unexpectedly found that, in addition to its previously reported distribution as an integral membrane protein [29], MALL localized to liquid-like PML NB biomolecular condensates. During mitosis, MALL accumulated in solid-like condensates around the spindle but, when in excess, the condensates mis-localized, altered the distribution of the nuclear proteins emerin LAP2β and BAF, and caused nuclear aberrations, which are a hallmark of cancer cells [32]. The presence of the MALL proteolipid in these two distinct environments —membranes and liquid-like condensates— and its differential detection in one place or the other depending on the principle —crosslinking or denaturation— of the method used for cell fixation suggest that MALL adopts two different conformations depending on its physical environment within the cell.

## Results

### MALL distributes in membranes and in the nuclear interior

**Fig. 1:**
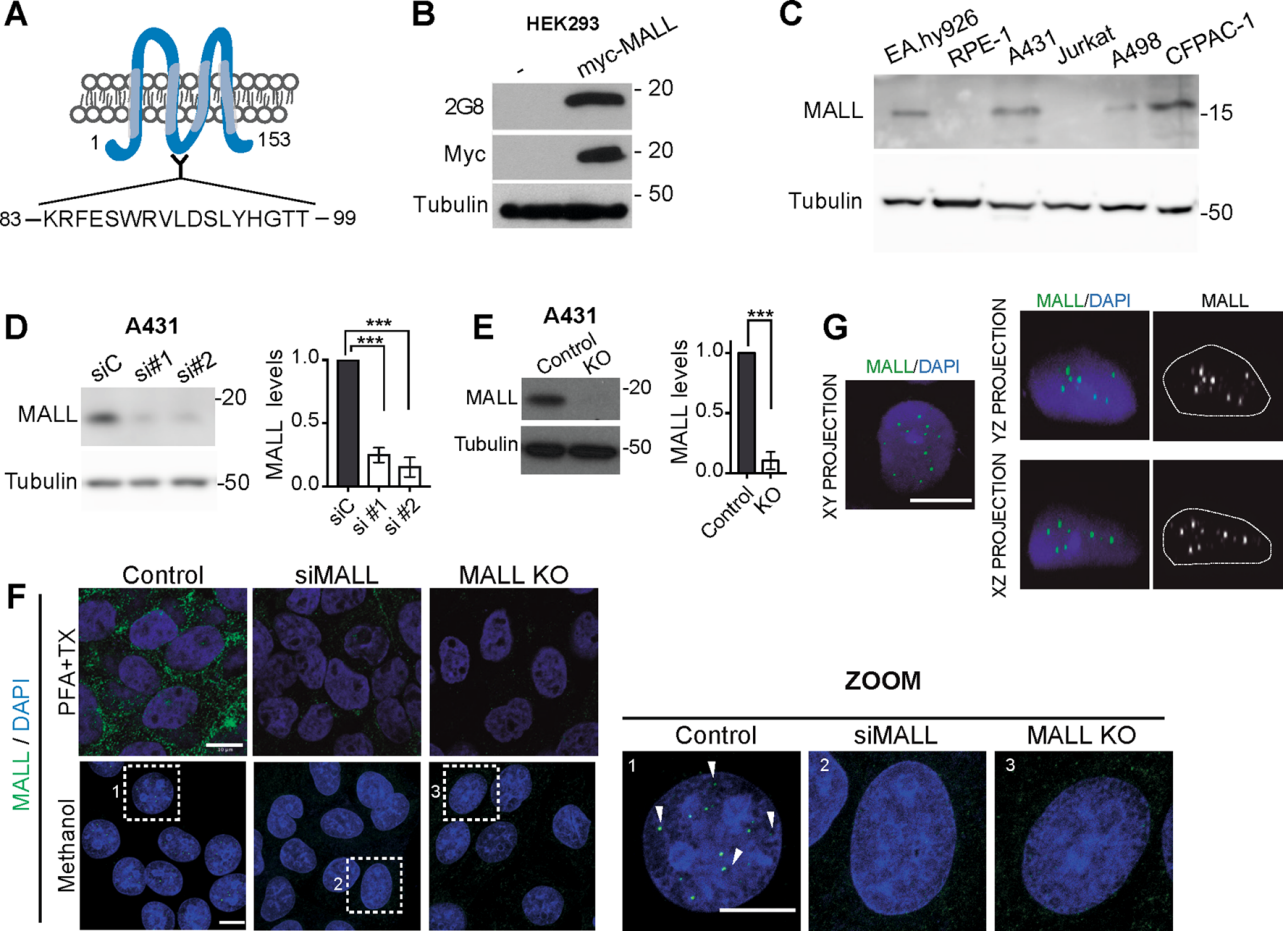
MALL distributes in membranes and nuclear dots. **A** Membrane-tetra-spanning structure of MALL, indicating the peptide used to generate antibodies to MALL. **B** HEK-293 cells were transfected or not with myc-tagged MALL and immunoblotted with 2G8 mAb and anti-myc antibodies. **C** Immunoblotting of extracts from different cell lines with mAb 2G8. **D** A431 cells were transfected with either control siRNA (siC) or two siRNAs (si#1 and si#2) targeting MALL for 72 h. Cell extracts were immunoblotted with mAb 2G8 (left panel). MALL levels were expressed relative to those in control cells (right panel). **E** Control and KO MALL cells extracts were analyzed by immunoblotting with mAb 2G8 (left panel). MALL levels were expressed relative to those in control cells (righ panel). The amount of tubulin was used as a loading control in (B–E). **F** Control, MALL KD and MALL KO A431 cells were fixed and permeabilized with PFA and Triton X-100 (top panels) or cold methanol (bottom panels) and were stained with mAb 2G8. An enlargement of the boxed regions is shown. The arrowheads indicate the presence of MALL in dots in the inter-chromatin space. **G** XY, XZ and YX projections of a 3D reconstructed nucleus of A431 cells fixed with cold methanol and stained with mAb 2G8. Nuclei were stained with DAPI in (F, G). Data in (D, E) represent the mean ± SEM of three independent experiments (***, *p* < 0.001)

To characterize human MALL, we previously obtained a rat mAb to residues 118–128 [29]. The antibody worked poorly for immunoblotting and it was entirely unsuitable for use in immunofluorescence and immunoprecipitation analyses, which greatly hampered progress in our research on MALL. In an attempt to overcome this obstacle, we used the peptide corresponding to amino acids 83–99 (Fig. 1A) and succeeded in generating of a mouse mAb (mAb 2G8) that specifically recognized exogenous MALL in HEK293 cells expressing myc-tagged MALL, but not in control cells (Fig. 1B). This antibody detected a protein band of the predicted size (17 kDa) in EA.hy926 umbilical vein immortalized cells, A431 epidermoid carcinoma cells, A498 kidney carcinoma cells, and CFPAC-1 ductal pancreatic adenocarcinoma cells, but not in RPE-1 retinal epithelium cells and Jurkat T cells (Fig. 1C). The level of the 17 kDa protein greatly decreased in A431 cells expressing two different siRNAs designed to knockdown (KD) MALL expression (Fig. 1D), and the protein was undetectable in A431 cells whose *MALL* gene was knocked out (KO) using the CRISPR/Cas9 DNA editing system (Fig. 1E).

Consistent with the reported distribution of myc-tagged MALL in epithelial ECV304 cells [29], endogenous MALL was detected at the plasma membrane and internal membranes in A431cells fixed with paraformaldehyde (PFA) and permeabilized with Triton X-100. The staining disappeared in A431 KD and KO cells, confirming the specificity of the antibody (Fig. 1F, top panel). To our surprise, the staining of A431 cells fixed with cold methanol produced a nuclear dotted pattern. The dotted pattern was lost in MALL KD and MALL KO cells (Fig. 1F, bottom panel). Controls omitting the primary antibody or using a control mAb in cells fixed with methanol produced no staining (Fig. S1A). A pattern similar to that of A431 cells was found in EA.hy926 and CFPAC-1 cells (Fig. S1B), which are positive for endogenous MALL (Fig. 1C), but not in RPE-1 cells (Fig. S1B), which are negative (Fig. 1C). The number of dots (average 17–19) per nucleus was similar in all the MALL-positive cell lines examined (Fig. S1C). Biochemical fractionation confirmed the presence of MALL in membrane and nuclear fractions of A431 cells (Fig. S1D). 3D reconstruction analysis indicated that the nuclear dots correspond to internal nuclear structures and not to invaginations of the nuclear envelope (Fig. 1G and Video 1).

**Fig. 2:**
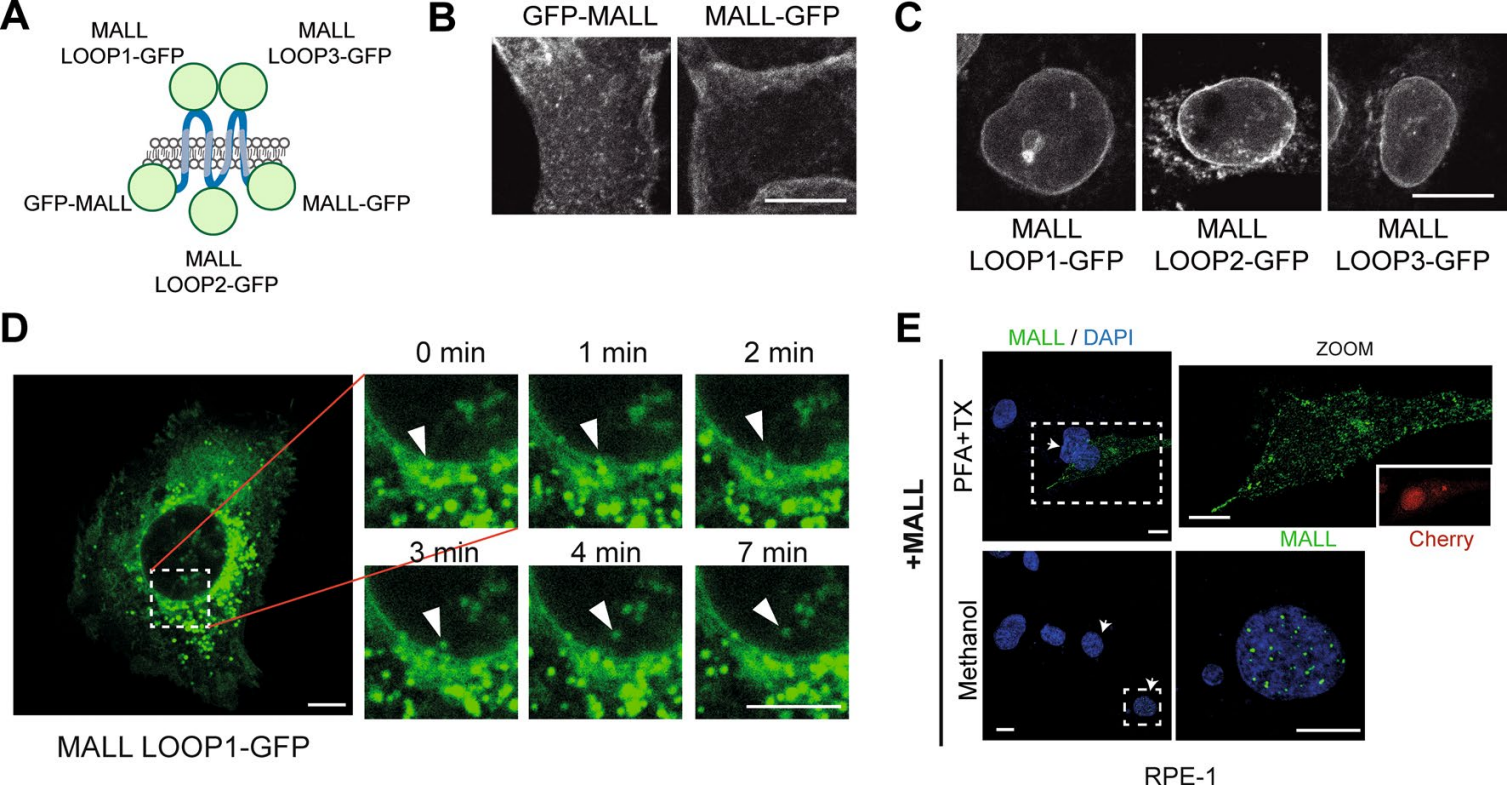
Distribution of exogenous MALL. **A** Scheme of the different chimeras of MALL and GFP used. **B–C** Distribution of the MALL chimeras with GFP at the N- or C-terminal end (B) or inserted in the loops (C). Only A431 cells with low levels of expression are shown. **D** Video-microscopic analysis of MALL LOOP1-GFP in cells with high levels of expression. MALL LOOP1-GFP was occasionally detected in small structures (arrowhead) that appear to traverse the nuclear envelope and enter the nucleus. **E** RPE-1 cells expressing exogenous intact MALL were fixed and permeabilized with either PFA plus Triton X-100 or with cold methanol. Cells were stained with mAb 2G8 and DAPI. Cherry fluorescence is shown in the inset as an example of identification of the transfected cells (top panels). Arrowheads indicate transfected cells. The enlargements correspond to the boxed regions. Scale bars, 10 µm

Given the unexpected presence of MALL in the nucleus, we tried to reproduce its distribution using different chimeras of MALL fused to GFP (Fig. 2A). The expression of GFP fused at the N- or C-termini of MALL recapitulated in live cells the distribution in membranes of endogenous (Fig. 1F) and myc-tagged MALL [29] observed in cells fixed with PFA (Fig. 2B). When GFP was introduced into any of the three loops of MALL, the chimeras labeled the nuclear envelope (MALL LOOP1-GFP) or the nuclear envelope and cytoplasmic membranes (MALL LOOP2-GFP and MALL LOOP3-GFP) in cells with low levels of expression (Fig. 2C), but were unable to reproduce the nuclear dotted pattern and the plasma membrane localization of endogenous MALL (Fig. 1F). At least in the case of MALL LOOP1-GFP, increasing levels of expression produced the appearance of labeled structures at the endoplasmic reticulum (ER) and the nuclear envelope (Fig. 2D). Although only sporadically, we were able to detect dots of MALL LOOP1-GFP entering the nucleus in the cells with highest level of expression (Fig. 2D and Video 2). These results indicate that the integrity of both N- and C-terminal ends is necessary for nuclear localization of MALL, and the integrity of the loops is required for MALL to enter the nucleus efficiently and to localize to the plasma membrane. Since none of the tagged-MALL chimeras reproduced the distribution of the intact molecule, we expressed intact MALL and the Cherry protein, which was used to visualize the transfected cell, simultaneously from a bi-cistronic mRNA. The expression of intact MALL in RPE-1 cells, which were negative for endogenous MALL (Fig. 1C and Fig. S1B), reproduced the plasma membrane localization of MALL in PFA-fixed cells and the dotted nuclear pattern in cells fixed with methanol (Fig. 2E). Together, Figs. 1, 2 and S1 show that endogenous MALL is present in membranes and nuclear dots.

### The nuclear dots containing MALL are PML nuclear bodies

**Fig. 3:**
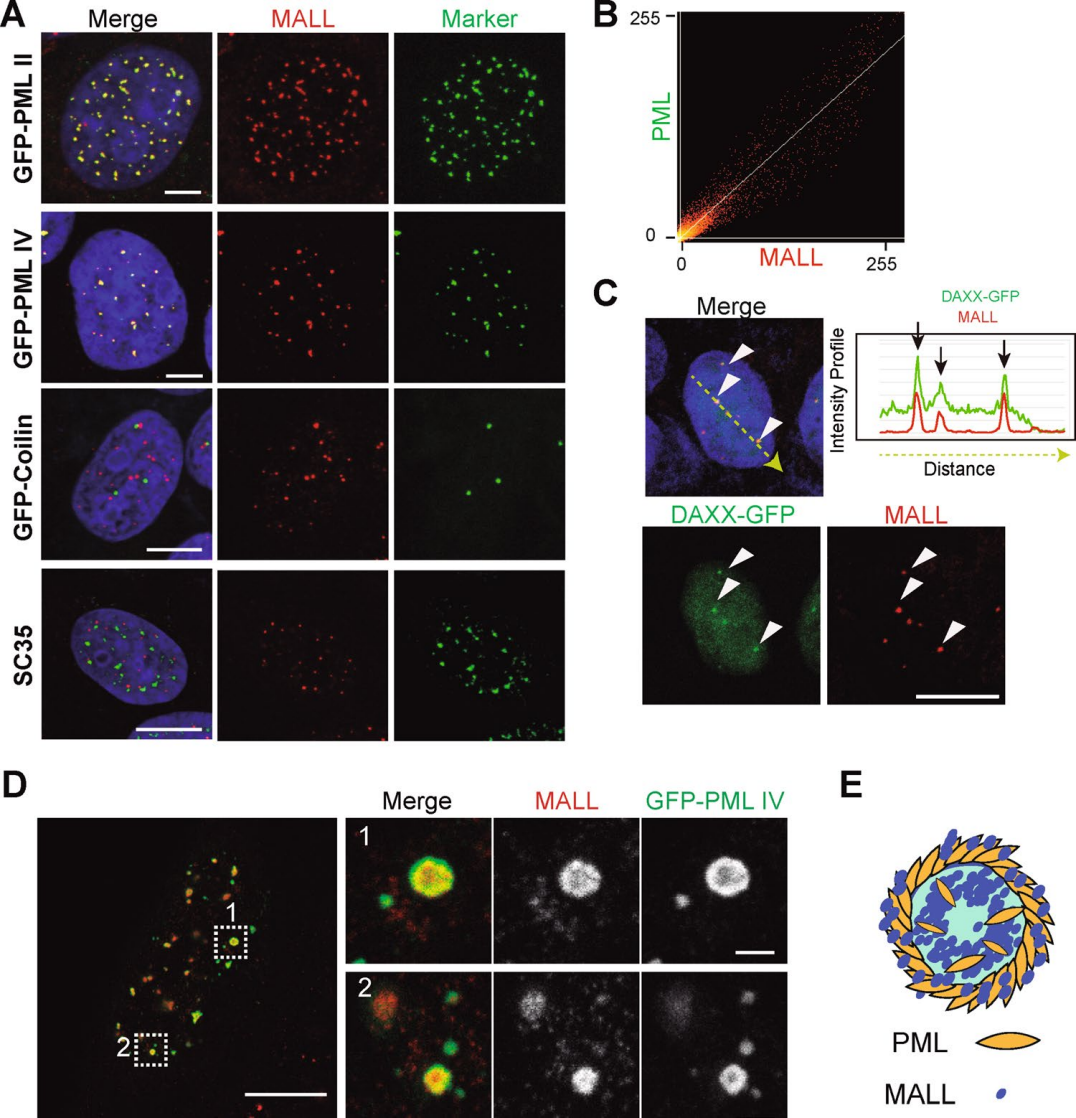
MALL localizes to PML NBs. **A** Co-localization study of endogenous MALL with GFP-PML II, GFP-PML IV, GFP-Coilin and endogenous SC35 in A431 cells. **B** Scatter plot of the co-localization between MALL and GFP-PML IV. **C** Cells expressing DAXX-GFP were fixed with methanol and stained for endogenous MALL with mAb 2G8. Arrowheads indicate puncta of co-localization. The graph shows the fluorescence intensity of MALL and DAXX-GFP measured along the green arrow. Scale bars (A, C), 10 µm. **D** STED images of PML NBs. Cells expressing GFP-PML IV were fixed with methanol and stained for endogenous MALL. Scale bars, 5 µm (panoramic), and 0.5 µm (enlargements). **E** Schematic of the distribution of PML and MALL in PML NBs

To identify the type of nuclear domains positive for MALL, we performed co-localization analyses of MALL with different endogenous or exogenous nuclear markers. MALL co-localized with the PML NB markers PML II-GFP and PML IV-GFP [33], but no with endogenous SC35 [34], which stains speckles [35], and coilin-GFP [36], which labels Cajal bodies [37] (Fig. 3A, B). No co-localization of MALL was found with nuclear invaginations, as visualized with lamina (GFP-lamin A, GFP-lamin B) [38] or inner membrane nuclear envelope—endogenous emerin [39] and lamin B receptor (LBR)-GFP [40]— markers (Fig. S2A). The PML NB marker DAAX-GFP [41] co-localized with MALL, confirming the presence of MALL in PML NBs (Fig. 3C). Unlike MALL, PML was immuno-localized in PML bodies also in cells fixed with PFA and permeabilized with Triton X-100 (Fig. S2B). Super-resolution stimulated emission depletion microscopy indicated that MALL was present predominantly at the periphery of PML NBs, partially overlapping the peripheral ring of PML, and also in the PML NB interior (Fig. 3D, E).

**Fig. 4:**
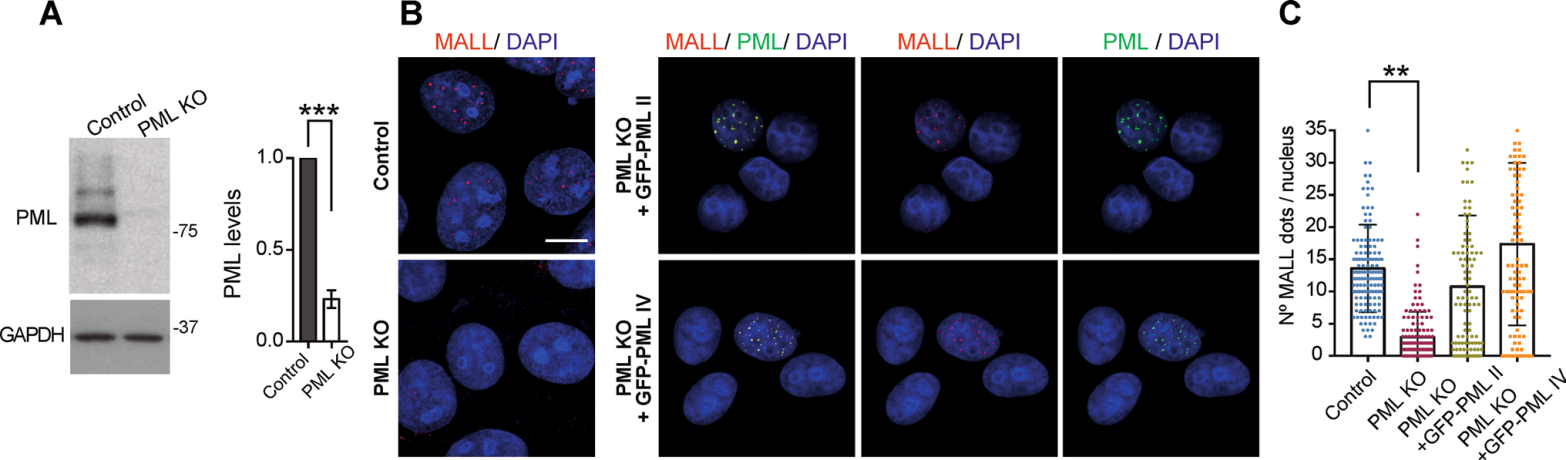
PML is necessary for the localization of MALL in the nucleus. **A** Control or PML KO A431 cells were immunoblotted for PML and GAPDH, which was used as a loading control (left panel). The levels of PML were quantified and expressed relative to those in control cells (right panel). **B** Control, PML KO cells, and PML KO cells expressing GFP-PML II and GFP-PML IV were fixed with methanol and stained for MALL and with DAPI. **C** The frequency of MALL dots per nucleus was quantified. More than 100 cells of each type were analyzed in the indicated types of cell. Each scored cell is represented by an individual dot in the graph. The mean ± SEM are shown. Three independent experiments were performed (***, *p* < 0.001). Scale bar, 10 µm

PML is essential for PML NB assembly [41]. Consistent with this role, the nuclear dotted pattern of MALL disappeared in PML KO A431 cells and was restored by exogenous expression of GFP-PML II and GFP-PML IV (Fig. 4A–C). As a control, we observed that the pattern was also lost in PML KD cells, but was unaltered in lamin A/C or emerin KD cells (Fig. S3A–C). MALL KO did not affect the number of PML NBs per cell (Fig. S3D), indicating that MALL is not necessary for PML NB assembly. This is consistent with the fact that most mammalian cells have PML bodies, although MALL is not ubiquitously expressed. For instance, RPE-1 cells, which do not express MALL endogenously (Fig. 1C), have PML NBs (Fig. S3E). In conclusion, Figs. 3 and 4 show that the nuclear pattern of MALL observed in methanol-fixed cells corresponds to PML NBs.

**Fig. 5:**
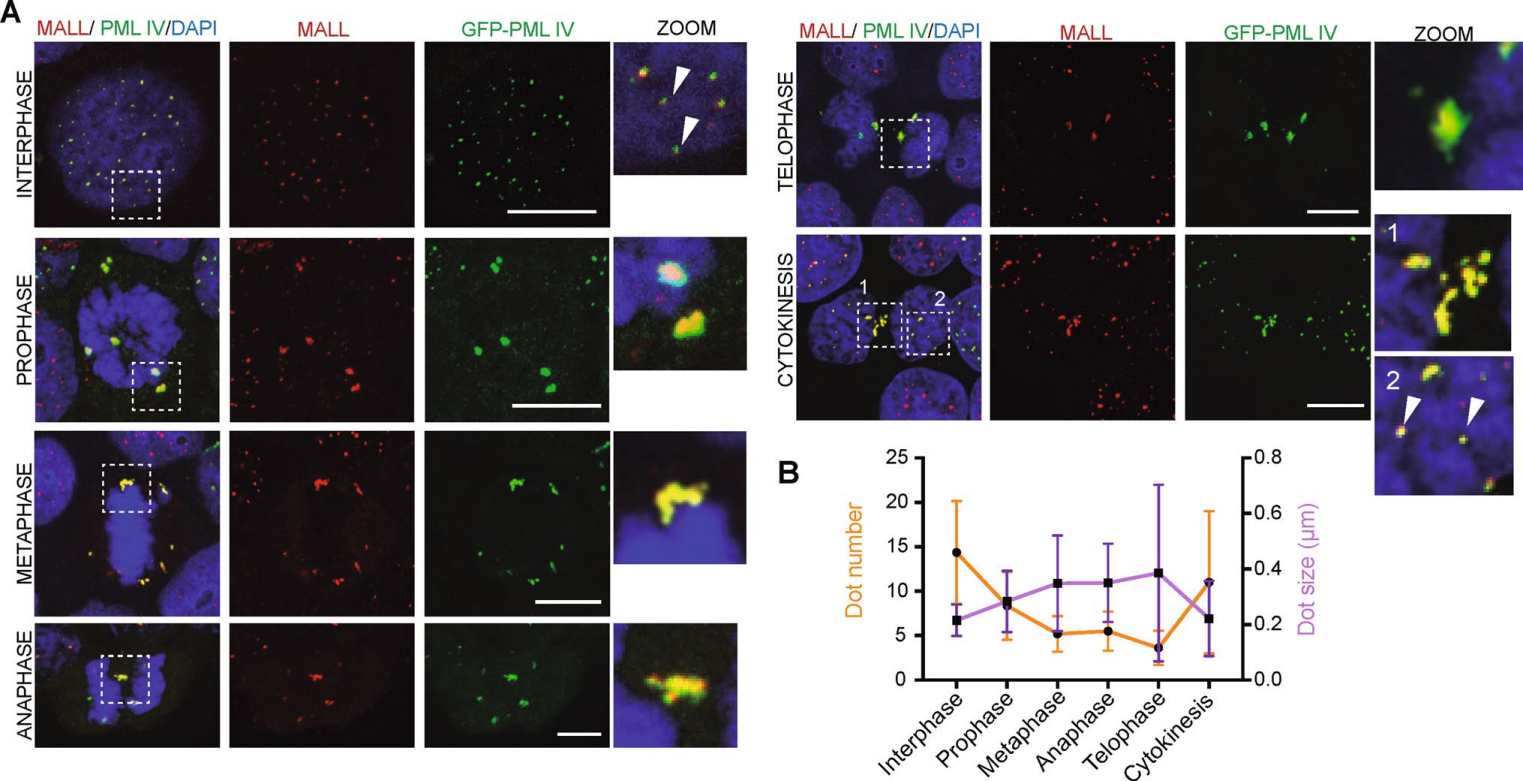
Distribution of MALL in mitotic cells. **A** A431 cells expressing GFP-PML IV were fixed with methanol and stained for MALL and with DAPI. The images correspond to cells in the indicated phase of mitosis. An enlargement of the boxes is shown. The arrowheads (interphase and cytokinesis panels) indicate the presence of MALL in the inter-chromatin space. **B** Quantification of the size (purple line) and frequency of MALL-positive dots per cell (yellow line) during mitosis. > 25 cells were analyzed for each phase. Three independent experiment were performed. Error bars represent the SEM. Scale bars, 10 µm

Unlike other compartments, PML NBs are not fragmented or disassembled after entry into mitosis, but rather they transform into a few, large, solid-like aggregates that distribute asymmetrically around of and, in a small proportion, associated with the metaphase chromosomes, and at the mitotic poles [42]. To investigate the distribution of MALL during cell division, we compared its distribution with that of GFP-PML IV. Since the chimeras of MALL and GFP did not reproduce the localization of the endogenous protein in PML NBs (Fig. 2 B, C), we reconstructed the process from still images obtained from methanol-fixed cells. We found that endogenous MALL and GFP-PML IV co-localized in the same aggregates throughout mitosis, and progressively incorporated into the nucleus, occupying inter-chromatin space once the nucleus has formed (Fig. 5A). The number of MALL-positive structures decreased while their size increased during mitosis and began to recover their interphase values during cytokinesis (Fig. 5B). The results presented in Fig. 5 indicate that, unlike DAAX or SP100 [42], MALL aggregates with PML during mitosis.

### Excess of MALL causes nuclear aberrations

**Fig. 6:**
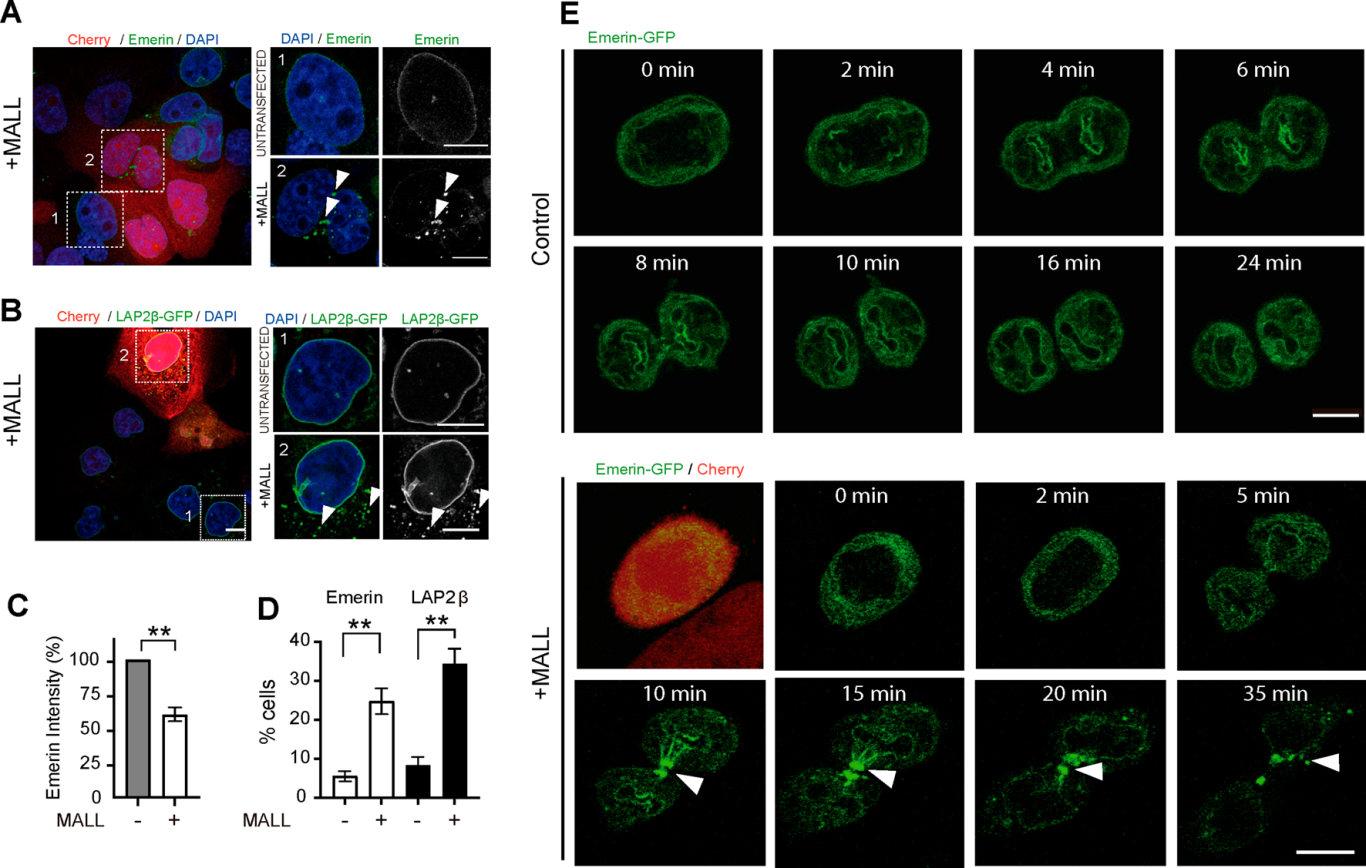
MALL overexpression alters the distribution of the LEM domain-containing proteins emerin and LAP2β. **A–D** Intact MALL was overexpressed using the bi-cistronic construct in control (A) or A431 cells expressing LAP2β-GFP (B), and were stained for endogenous emerin (A) or analyzed for LAP2β-GFP (B). Boxes 1 and 2 correspond to un-transfected or transfected cells, respectively, which were identified by the absence or presence of Cherry expression, respectively. Box 2 shows the presence of cytoplasmic aggregates (arrowheads). The histograms illustrate the levels of emerin at the nuclear envelope (C) and the percentage of cells with aberrant aggregates of emerin and LAP2β–GFP (D). Data are summarized as mean ± SEM of three independent experiments (120 un-transfected and 150 transfected cells were analyzed in the case of emerin; 150 control cells and 90 transfected cells were analyzed in the case of LAP2β) (**, *p* < 0.01). **E** Sequential frames of emerin–GFP in control cells (top panels) and in cells overexpressing exogenous intact MALL (bottom panels). The Cherry channel served to identify the cells overexpressing MALL. Note the accumulation of emerin–GFP in the intercellular bridge (arrowhead) in the case of MALL overexpression. Scale bars, 10 µm

The cancer microarray Oncomine^™^ database (www.oncomine.org) indicates that *MALL* is overexpressed in some types of cancer, especially in pancreatic cancer, compared with the content in normal tissue (Fig. S4A). Importantly, the data in the Human Protein Atlas database (www.proteinatlas.org) indicate that *MALL* overexpression in pancreatic cancer is correlated with unfavorable outcome (Fig. S4B). Bioinformatic analysis revealed that, although it was not statistically significant, there is a trend between high levels of MALL expression and aneuploidy in pancreatic cancer (Fig. S4C), suggesting that MALL overexpression could contribute to cell malignancy by promoting nuclear aberrations. To investigate the effect of MALL excess, we expressed intact MALL using the bi-cistronic construct and analyzed the distribution of emerin and LAP2β, which are two integral proteins of the inner membrane of the nuclear envelope that contain a LAP2-emerin-MAN1 (LEM) domain [39]. MALL overexpression decreased the levels of endogenous emerin from the nuclear envelope, causing it and LAP2β-GFP to disperse in cytoplasmic aggregates in a fraction of the transfected cells (Fig. 6A–D). Video-microscopic analysis indicates that whereas emerin incorporated correctly in the nuclear envelope of control cells, it remained accumulated at the ends of the intercellular bridge that connect the sister cells during cytokinesis in the cells overexpressing MALL (Fig. 6E, Video 3).

**Fig. 7:**
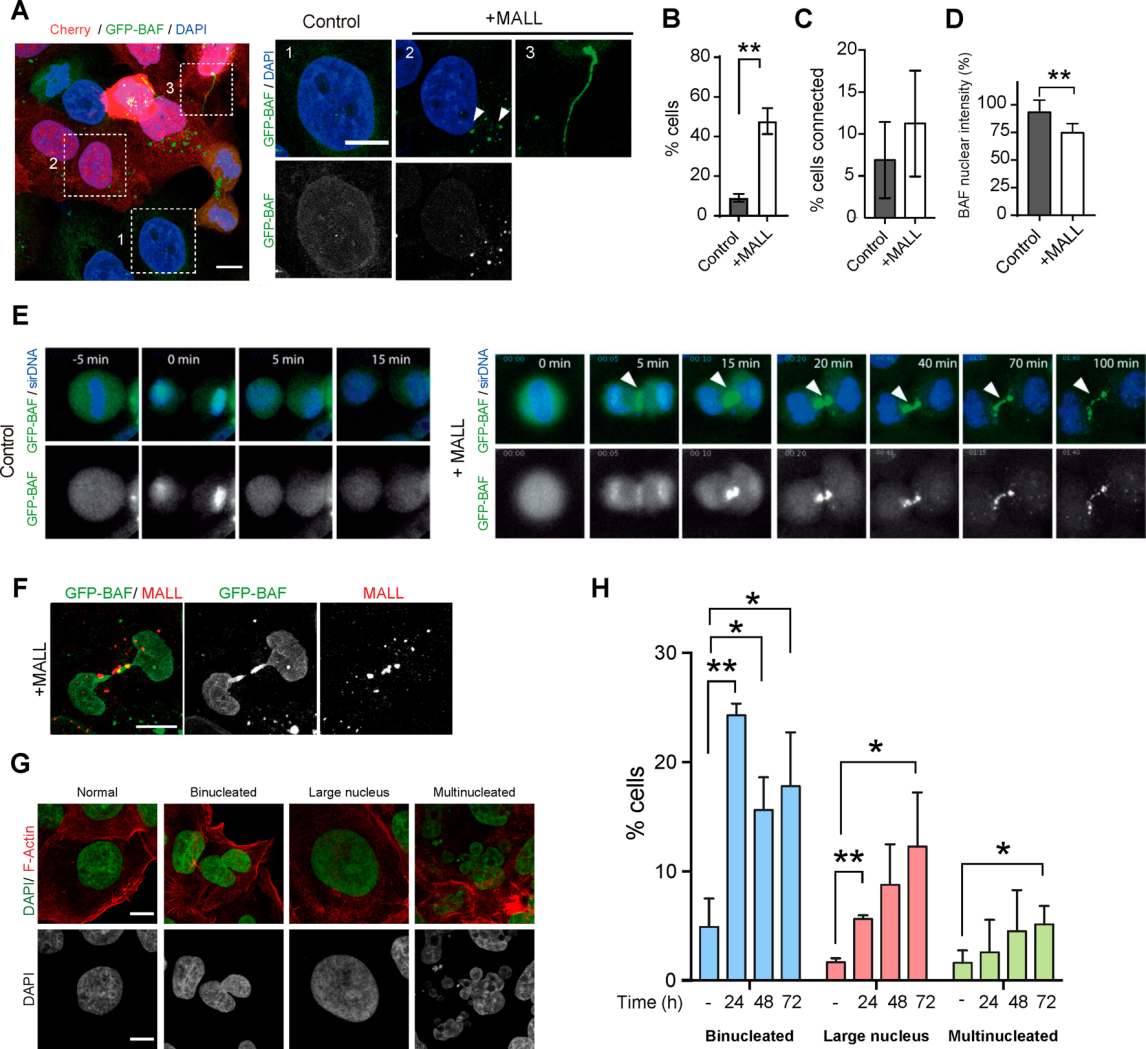
MALL overexpression alters GFP-BAF localization and causes nuclear aberrations. **A** MALL was overexpressed in A431 cells expressing GFP-BAF and the distribution of BAF-GFP was examined. Note the presence of GFP–BAF cytoplasmic structures (box 2) and intercellular bridges (box 3) in cells overexpressing MALL. The right panels show enlargements of box 1, which corresponds to a control cell, and of boxes 2 and 3 that correspond to transfected cells, as revealed by Cherry fluorescence. **B**-**D** The histograms represent the percentage of cells with aberrant cytoplasmic structures of GFP–BAF (B), the number of cells connected by bridges containing GFP–BAF (C), and the levels of nuclear GFP–BAF in cells overexpressing MALL relative to control cells (D). 300 control cells and 148 MALL-overexpressing cells were analyzed. **E** Video-microscopic analysis of the dynamics of GFP–BAF in control (left panels) and MALL-overexpressing cells (right panels). Note the accumulation of BAF–GFP within the intercellular bridge (arrowhead). **F** Cells expressing GFP–BAF were fixed with methanol and stained for MALL. Note the presence of MALL aggregates within the intercellular bridge. **G** Representative images of the various nuclear phenotypes observed in MALL-overexpressing cells. Cells were stained for F-actin and with DAPI. **H** The percentage of MALL-overexpressing cells with the indicated aberrant nuclear phenotype was quantified at the indicated times post transfection. 184 cells control cells and 169 cells overexpressing MALL were analyzed. The mean ± SEM are shown. Three independent experiments were performed in (B–D, H) (*, *p* < 0.05; **, *p* < 0.01). Scale bars, 10 µm

Barrier-to-auto-integration factor (BAF) interacts with LEM domain-containing proteins, and is associated with the chromosomes at the anaphase-telophase transition [43–45]. BAF is required for the incorporation of emerin and probably other membrane-bound LEM proteins into the reforming nuclear envelope [43]. Since emerin and LAP2β were dispersed in MALL-overexpressing cells, we investigated whether the nuclear targeting of BAF was also affected. We found MALL overexpression produced the accumulation of BAF into cytoplasmic structures and within bridges connecting pairs of cells, and caused a slight reduction in the levels of BAF in the nucleus (Fig. 7A–D). The bridges were identified by video-microscopic analysis as unresolved cyto-kinetic bridges (Fig. 7E, Video 4). In addition to emerin aggregates (Fig. 6D, Video 3), MALL aggregates accumulated within the bridges, but they did not co-localize with BAF (Fig. 7F). No effect on BAF distribution was observed in MALL-depleted cells (Fig. S5A–C).

BAF connects LEM domain-containing transmembrane proteins to chromatin [43, 45–47], but this association is not required for nuclear assembly [48]. BAF also connects distant chromatin sites into a compact network that impedes the access of ER membranes, which otherwise would pack chromosomes into different nuclei, producing multinucleated cells [48]. Abnormal formation of cyto-kinetic bridges is formed when a chromosome, or part of it, is stretched between the two separating masses of cellular DNA at the final stage of cell division because of chromosome segregation errors that occurred in earlier stages. If the problem is solved, cytokinesis occurs normally, but if it persists, the cyto-kinetic bridge usually regresses and, as consequence, the process ends in the formation of a bi-nucleated cell. Then, bi-nucleated cells may arrest, or may divide either with a bipolar spindle, which yields two tetraploid cells with one large nucleus, or with a multipolar spindle, which produces cells with multiple, small nuclei [49]. When MALL-overexpressing cells were analyzed, we observed all these aberrant nuclear phenotypes (Fig. 7G), bi-nucleated cells being the most abundant subset at 24–72 h post transfection, with a progressive increase of the fractions of mono-nucleated cells with a large nucleus and of multinucleated cells over that period (Fig. 7H). In conclusion, excess MALL produces an accumulation of BAF within cyto-kinetic bridges and, subsequently, the appearance of cells with nuclear aberrations.

## Discussion

The *PML* gene, which was originally identified as part of a recurrent chromosomal translocation in the great majority of patients with acute PML, is the scaffold protein of PML NB condensates [50, 51]. PML NBs have been involved in a large repertoire of functions, such as transcription regulation, chromatin association and DNA repair [51]. MALL is a proteolipid with a membrane-tetra-spanning topology similar to that of the PLP, PLLP and MAL proteolipids [29]. In this work, using a new mAb to MALL and PFA or cold methanol as methods of cell fixation, we have differentially identified MALL in membranes and PML NB biomolecular condensates, respectively. Similar to PML, and unlike the rest of PML NB components analyzed [42, 52], MALL redistributed to PML aggregates during mitosis. Overexpression of MALL perturbed the distribution of the LEM domain-containing proteins emerin and LAP2β, and that of the DNA-binding protein BAF, which interacts with LEM proteins, during mitosis, leading to the formation of aberrant nuclei. This finding might be related to the possible contribution of MALL to the acquisition of a malignant cell phenotype.

Myc-tagged MALL was reported to localize to the plasma membrane and cytoplasmic membranes in epithelial ECV304 cells [29]. This distribution was reproduced for the endogenous protein with the newly generated anti-MALL mAb 2G8 in epithelial A431 cells fixed with PFA. However, when these cells were fixed with cold methanol, the antibody revealed the presence of MALL in the nucleus, forming part of PML NB condensates. Cell fixation with PFA involves extensive protein crosslinking by covalent bond formation, whereas methanol dehydrates cells and disrupts hydrophobic interactions, causing protein denaturalization and precipitation. Differential protein localization using one or other chemical fixative is rarely observed for other proteins. For instance, in ciliated cells, the intra-flagellar transport subunit IFT20 is detected exclusively in the Golgi in PFA-fixed cells, and at the basal body/centrosome in the case of methanol fixation [53]. A plausible explanation for this type of observation is the presence of the protein in distinct environments and/or conformations that allows or not the access of the antibody or the recognition of the epitope in a fixative-dependent manner. Our results indicate that MALL is simultaneously present in the cell in two distinct environments, membranes and liquid-like biomolecular condensates. Obviously, MALL must adopt two distinct conformations to fit them.

The membrane-tetra-spanning PLP and PLLP proteolipids can be converted in vitro into a water-soluble form by gradually replacing the organic solvent by water [9, 18–20]. This conversion produces a profound structural change in such a way that, when they are inserted into liposomes, their structure is consistent with the presence of transmembrane segments and short extra-membranous loops, whereas in an aqueous environment, they adopt a conformation with maximal exposure of its scarce hydrophilic residues and a large hydrophobic core [9, 21]. Our results suggest that the MALL proteolipid exists in the cell in two conformations: one as an integral membrane protein, and another, probably similar to that of water-soluble PLP and PLLP, that is compatible with the aqueous environment of the PML NB condensates. Cell fixation with PFA, but not with methanol, allows detection of the membrane-embedded MALL conformer, whereas the opposite is true for the conformer in PML NB condensates*.*

**Fig. 8:**
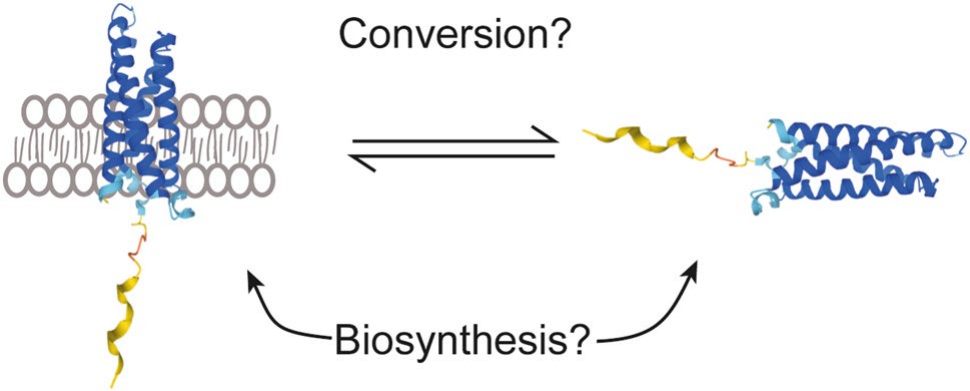
Schematic showing how MALL might acquire a structure compatible with membranes or with liquid-like biomolecular condensates. MALL could be diverted during biosynthesis to adopt one or the other conformation. Alternatively, the existence of the two forms of MALL might result from conformational conversion

The acquisition of a structure compatible with membranes or with liquid-like biomolecular condensates could be a direct event during MALL biosynthesis or the result of a conformational conversion (Fig. 8). In the first case, MALL biosynthesis must be assisted by different machinery of folding to adopt one or another conformation. In the second case, the transition from one environment to another must be accompanied by the simultaneous conversion of the membrane-tetra-spanning structure of MALL to one compatible with the membrane-less PML NB condensates, which implies MALL de-lipidation and a dramatic conformational change, also assisted by specialized machinery. As an integral membrane protein, the tetra-spanning conformation must be acquired in the ER. Regardless of which of the two scenarios is true, the different fate and conformations raise the intriguing questions of where and how MALL acquires the condensate-compatible conformation. Since endogenous MALL was detected within the nucleus, but not in the cytoplasm in methanol-fixed cells, the acquisition of this conformation should take place rapidly in the nuclear envelope or adjacent ER membranes. Supporting this possibility, we observed that, although the presence of MALL in PML NBs was not reproduced using GFP-tagged MALL, the chimeras with GFP at the MALL loops, especially MALL LOOP1-GFP, localized to the nuclear envelope at low levels of expression and also, when the levels of expression were high, to proximal ER-associated profiles. We also sporadically observed that some of these profiles entered the nucleoplasm. The GFP insertion (256 amino acids), which is very bulky compared with the size of first loop (17 amino acids) of MALL, and larger than the entire MALL molecule (153 amino acids), probably restrains the conformational flexibility of MALL, making the acquisition of the condensate-compatible conformation or the transition to the nuclear interior very inefficient, with the consequent accumulation of the chimera into the nuclear envelope and adjacent ER membranes. The observation that endogenous MALL was undetectable in PML KO cells fixed with methanol indicates that PML is necessary for MALL to acquire the condensate-compatible conformation and that it could assist MALL during this process.

Liquid-like biomolecular condensates can mature to form gels or solidify into aggregates [54]. During mitosis, instead of being fragmented into smaller structures to facilitate its inheritance and subsequent reassembly after separation of the sister cells, PML transitions from PML NBs liquid-like biomolecular condensates into a few, large condensates with a solid-like state [42, 52]. These condensates contain de-SUMOylated PML, and are devoid of DAAX, SP100 and probably most PML NB components, due to the inability of de-SUMOylated PML to recruit proteins containing SUMO-interacting motifs [42, 52, 55]. Notably, MALL co-localized with PML in the solid-like aggregates in methanol-fixed cells, suggesting that MALL is able to transition from a liquid-like to a solid-like condensate. The loss of the latter type of condensate and the increased number of MALL-containing PML NBs at the end of mitosis suggest that this transition is reversible, as is the case for that of PML, to allow MALL to be recycled to PML NBs in the newly formed nuclei. Therefore, MALL, in addition to being embedded in cellular membranes, transits with PML from liquid-like PML NB condensates in interphase to solid-like condensates during mitosis.

The affinity of MAL and PLP for condensed membranes was confirmed using giant plasma membrane vesicles (GPMVs) [13], which are spherical structures that segregate compact and fluid membrane subdomains in opposing hemispheres [56, 57]. MALL, however, showed minimal preferences for GPMV condensed membranes, although it partitioned efficiently into DRMs [13]. GPMVs are closer to physiological membranes than are DRMs [58]. However none of them is a *bona fide* condensed native membrane because important factors (e.g., lipid asymmetry, assembled cytoskeleton, protein–protein interactions) are disrupted by the GMPV isolation procedure, and extensive artifactual membrane reorganization occurs during DRMs isolation [58]. Regardless of its presence in membranes and PML NB condensates in the cell, MALL partitioned almost exclusively into the organic phase or the DRM fraction when cells were extracted with an organic solvent or a nonionic detergent, respectively [29]. This behavior probably reflects an intrinsic tendency of MALL to partition into the organic solvent or DRM lipids during the respective extraction procedure, independently of its environment in the cell.

Previous studies of MALL gene expression by northern blot [29] and quantitative RT-PCR analyses (www.proteinatlas.org) indicated that *MALL* is expressed only in a restricted range of cell lines. Consistent with this finding, the analysis of the *MALL* mRNA levels in 51 human tissues showed enhanced expression in the esophagus, intestine and lung, and low or undetectable levels of expression in the other tissues analyzed (www.proteinatlas.org). These data, together with the analysis of single-cell RNA specificity using primary single-cell types, suggest that *MALL* is expressed only in a restricted pattern of cell types and tissues (www.proteinatlas.org). Consequently, MALL is dispensable in most cells. If the function of MALL is important, it might be assumed by proteins with related primary structure and or/similar characteristics, for instance members of the MARVEL domain-containing superfamily to which the MAL protein family belongs to [10, 59]*.* Oncogenic transformation appears to induce or upregulate MALL expression in some tissues and to downregulate it in others (Fig. S4A). In the case of pancreatic cancer, MALL RNA is expressed in large excess compared with normal tissue, and its overexpression correlates with an unfavorable patient outcome (Fig. S4B). In cultured cells, we observed that MALL overexpression produced the accumulation of MALL and emerin in solid-like aggregates in or near the cyto-kinetic bridge. Under these conditions, BAF, which normally connects LEM proteins with chromatin, accumulates along the bridge, leading to the formation of aberrant nuclei, which are a hallmark of cancer [32]. Therefore, the alteration produced by an excess of MALL could contribute to pancreatic cancer by producing aberrant nuclei (Fig. S4C).

Although the function of MALL in PML NB condensates is currently unknown, given its dual protein and lipid-like properties and structural flexibility, a speculative possibility is that it relaxes surface tension at the interface between the aqueous milieu of the nucleoplasm and PML NB condensates, thereby helping to prevent their collapse. It is possible that other membrane-tetra-spanning proteolipids can adopt distinct conformations for biological processes that generate high tension, for instance during the formation of the fusion pore [60]. In the case of MALL, the use of mAb 2G8 as a tool combined with distinct methods of cell fixation allowed detection of MALL in different environments and conformations. Detection of different conformations in other proteolipids requires tools —for instance, an appropriate antibody— that might not be available, and experimental conditions —for instance cell fixation methods— that need to be empirically established.

In conclusion, the MALL proteolipid distributes in the cell in membranes and PML NBs biomolecular condensates, it being possible to distinguish the two pools with our novel anti-MALL mAb and the method of cell fixation used. The presence of MALL in liquid-like biomolecular condensates requires the acquisition of a conformation very different from its tetra-spanning structure in membranes. Adoption of such a structure probably occurs in the nuclear envelope or adjacent ER membranes and requires PML expression. Excess MALL promotes aberrant nuclei and, consistent with the unfavorable prognosis of MALL overexpression in pancreatic cancer, might contribute to cell malignancy. This points to MALL as being a possible target for cancer treatment. The anti-MALL mAb 2G8 described in this study constitutes a unique tool for investigating the acquisition of the two alternative structures of MALL further, for analyzing its role in cancer, and for establishing pancreatic cancer prognosis. Our study establishes a link between proteolipids, membranes and biomolecular condensates, and lends experimental support to the abiding speculation that membrane-tetraspanning proteolipids are present in two distinct conformations in the cell.

## Materials and methods

### Generation of a monoclonal antibody to human MALL

The peptide KRFESWRVLDSLYHGTT, corresponding to amino acids 83–99 of human MALL was synthesized in an automated multiple peptide synthesizer (AMS 422, Abimed, Langerfeld, Germany) using the solid-phase procedure and standard Fmoc [*N*-(9-fluorenyl)methoxycarbonyl] chemistry [61]. After coupling to keyhole limpet hemocyanin, the peptide was used to immunize mice. Spleen cells from immunized mice were fused to myeloma cells and plated onto microtiter plates. The culture supernatants were screened by immunoblot analysis. The hybridoma clone 2G8, which secretes a mAb to human MALL, was isolated after several rounds of screening and sub-cloning, and was used to produce culture supernatants containing the antibody.

### Antibodies and other reagents

The sources of the antibodies to the different markers were as follows: emerin (mouse mAb IgG2b, used at 1/250 for immunofluorescence analysis (IF) and at 1/500 for western blot (WB); sc-25284); PML (mouse mAb IgG1; used at 1/250 for IF and 1/500 for WB; sc-377390); lamin A/C (mouse mAb; used at 1/250 for IF and WB; sc-7292) were obtained from Santa Cruz Biotechnology, Inc; GAPDH (mouse mAb IgG1; used at 1/20,000 for WB; AM4300) was from ThermoFisher Sci.; α-tubulin (mouse mAb IgG1; used at 1/500 for IF and 1/200 for WB; T6074) was from Sigma-Aldrich; caveolin-1 (mouse mAb IgG1, used at 1/2,000 for WB; 610,058) was from BD Biosci. The hybridoma producing antibodies to SC35 (mouse mAb IgG1, used at 1/50 for IF; CRL-2031) and myc (9E10) (mouse mAb IgG1, used at 1/500 for IF and WB; CRL-1729) were obtained from the American Type Culture Collection (ATCC). Secondary antibodies conjugated to Alexa Fluor-488, -555, -594 and -647 were from ThermoFisher Sci. 4′,6-Diamidino-2-phenylindole dihydrochloride (DAPI; 268,298) was from Merck, and SiR-DNA (CY-SC007) from Cytoskeleton Inc. Horseradish peroxidase-labeled secondary antibodies were from Jackson Immunoresearch Laboratories, Inc.

### Cell culture

A431 (CRL-1555), EA.hy926 (CRL-2922), A498 (HTB-44) and CFPAC-1 (CRL-1918), Jurkat (TIB-152) and RPE-1 (CRL-4000) cells were obtained from the ATCC. They were grown in DMEM (A431, EA.hy926, A498 and CFPAC-1), RPMI medium (Jurkat) or DMEM F12 (RPE-1), supplemented with 10% FBS, and were maintained at 37 °C in a humidified atmosphere of 95% air/5% CO_2_. Mycoplasma testing was regularly performed.

### DNA constructs, siRNAs and transfection conditions

The following plasmids were obtained from Addgene: DAXX-GFP (plasmid #119,021) [62], emerin pGFP-C1 (plasmid #61,993) [63], pBABE-puro-GFP-wt-lamin A (plasmid #17,662) [64], pEGFP-coilin (plasmid #36,906) [36]. The EGFP-PML II and EGFP-PML IV constructs [33] and the LAP2β construct [65] were kind gifts from T. Fujimoto (Nagoya University, Tokyo, Japan) and J. Ellenberg (EMBL, Heidelberg, Germany), respectively. The EGFP-lamin B1 plasmid was made from mCherry-lamin B1–10 (Addgene plasmid #55,069). The construct expressing myc-tagged-MALL was described previously [29]. The N- and C-terminal fusions of MALL and GFP were generated by inserting the MALL coding sequence in-frame with GFP using the EGFP-C1 and EGFP-N1 vectors, respectively. The DNA fragments encoding MALL proteins with GFP inserted into the loops (MALL LOOP-GFP constructs 1–3) were generated by site-directed mutagenesis by overlap extension using PCR [66] and were cloned into the pCR3.1 mammalian expression vector (ThermoFisher Sci.). A DNA construct in the pcDNA3.1 mammalian expression vector (ThermoFisher Sci.) consisting of the GFP or Cherry coding sequence and the intact MALL coding sequence separated by the foot-and-mouth virus internal ribosome entry site sequence from pBIC [67] (a kind gift from Dr. E. Martínez-Salas, CBMSO, Madrid, Spain) was used to express Cherry and MALL simultaneously from a bicistronic mRNA transcribed from the cytomegalovirus early promoter. DNA constructs were transfected using an ECM 600 (BTX, San Diego, CA) Electroporator, with 200 V; 960 μF and 480 Ω. For stable expression, transfected cells were selected with 1 mg/ml G-418, and individual cell clones were screened by immunofluorescence and immunoblot analyses. For siRNA-mediated MALL KD, we used the siRNA duplexes 5’-AAGAAGGCGAAAGGGAUGGTT-3’ (siMALL#1) and 5’-AAGGACGUUCUGUUAAUCCTT-3’ (siMALL#2). siControl (5’-UAGCGACUAAACACAUCAA-3’) was used as non-targeting siRNA. Silencer^™^ Pre-Designed siRNAs (ThermoFisher Sci.) were used for KD of PML #AM16708 (5’-GAUGCAGCUGUAUCCAAGATT-3’), emerin #4,392,420 (5’-GCUUUACUCUACCAGAGCATT-3’), and lamin A/C #4,390,824 (5’-CCAAAAAGCGCAAACUGGATT-3’. Cells were transfected with 20 nM of the indicated siRNAs using Lipofectamine 2000 following the manufacturer’s (ThermoFisher Sci.) recommendations.

### Generation of KO cell clones

For CRISPR/Cas9 gene editing, the MALL and PML sequence were analyzed using the Breaking-Cas tool (http://bioinfogp.cnb.csic.es/tools/breakingcas), and the selected target sequences 5´ CGCCCCGTCCGACGTGCCCTCGG 3´ and 5´ CCGAGGGCACGTCGGACGGGGCG 3´ in the case of *MALL*, and 5’ GTCGGTGTACCGGCAGATTG 3´and 5’ TCTCGAAAAAGACGTTATCC 3’ in the case of *PML,* were inserted into the pSpCas9 (BB)-2A-GFP (PX458) plasmid (Addgene plasmid #48,138). GFP-positive cells were sorted 48 h after transfection and plated onto Petri dishes. Individual cell clones were screened by immunofluorescence and immunoblot analyses.

### Subcellular fractionation

Cells were scrapped into 500 μl of hypotonic buffer (10 mM Tris–HCl, pH 7.5; 10 mM NaCl; 3 mM MgCl_2_). After 15 min at 4 ºC, cells were broken by passing them through a 22G-needle. Nuclei were sedimented by low-speed centrifugation and the supernatant (S1), which contains membranes and cytoplasm, was incubated with 0.2% Triton X-100. After 15 min, membrane (P2) and cytosolic (S2) fractions were isolated by centrifugation at 100,000 x*g* for 1 h. Nuclei were extensively washed, were placed on the top of a 0.8 M sucrose cushion, and were centrifuged at 10,000 x*g* for 20 min. After SDS-PAGE, aliquots from the different fractions were analyzed by immunoblotting with the indicated antibodies.

### Conventional and super-resolution stimulated emission depletion microscopy, and live-cell imaging

Cells grown on glass coverslips were prepared for antibody staining by two different procedures: (1) with 4% paraformaldehyde for 20 min at room temperature, followed by incubation with glycine in PBS for 5 min to quench the aldehyde groups, and permeabilization with 0.2% Triton X-100 at 4 ºC for 10 min; or (2) with cold methanol for 5 min. Afterward, cells were treated with 3% bovine serum albumin in PBS for 10 min and then incubated with the primary antibody and DAPI. After 1 h at room temperature, cells were washed and incubated with the appropriate fluorescent secondary antibody. For double-labeling experiments, the same procedure was repeated for the second primary antibody. Cells were mounted in coverslips using ProLong Gold antifade reagent (ThermoFisher Sci.). Confocal images were captured using a Zeiss LSM800 confocal microscope with a 63x/1.4 oil objective. For super-resolution microscopy, stained cells were mounted with Prolong Diamond antifade reagent (ThermoFisher Sci.). Images were obtained using a confocal multispectral Leica TCS SP8 system equipped with a 100x/1.40 oil objective. The images were acquired using 6X zoom. Live-cell imaging was performed on cells grown in µ-slide 8 well chambers (ibidi GmbH) under a Nikon A1R + microscope with a 60x/1.2 water objective. Brightness and contrast were optimized with ImageJ (National Institutes of Health) and Photoshop (Adobe Systems). Quantifications were carried out using ImageJ.

### The cancer genome atlas program (TCGA) data analysis

Clinical and mRNA expression data from 176 patients with pancreatic cancer were extracted from the TCGA-PAAD project (https://portal.gdc.cancer.gov/projects/TCGA-PAAD). The aneuploidy scores were obtained from [68]. The survival curve was plotted using R package survminer (r-projcet.org).

### Data analysis

Statistical analysis and graphs were performed using Prism 6 software (GraphPad). Statistical significance was assessed with a two-tailed Student’s unpaired t test. All data are presented as the mean ± SEM and a value of *p* < 0.05 was considered statistically significant. *, **, ***, correspond to values of *p* < 0.05, *p* < 0.01, and *p* < 0. 001, respectively.

### Supplementary Information

Below is the link to the electronic supplementary material.

Supplementary file1 (AVI 775 KB)

Supplementary file2 (AVI 486 KB)

Supplementary file3 (AVI 2614 KB)

Supplementary file4 (AVI 4101 KB)

Supplementary file5 (PDF 9401 KB)

## Data Availability

The datasets generated during and/or analyzed during the current study or the materials used are available from the corresponding author upon request.
