# Supplementary material for: MALL, a membrane-tetra-spanning proteolipid overexpressed in cancer, is present in membraneless nuclear biomolecular condensates
Source: Cell Mol Life Sci. 2022 Apr 10;79(5):236. doi: 10.1007/s00018-022-04270-w (PMC8995265; doi:10.1007/s00018-022-04270-w)
Supplement: Supplementary file 5 — Supplementary file5 (PDF 9401 KB) [file 18_2022_4270_MOESM5_ESM.pdf]

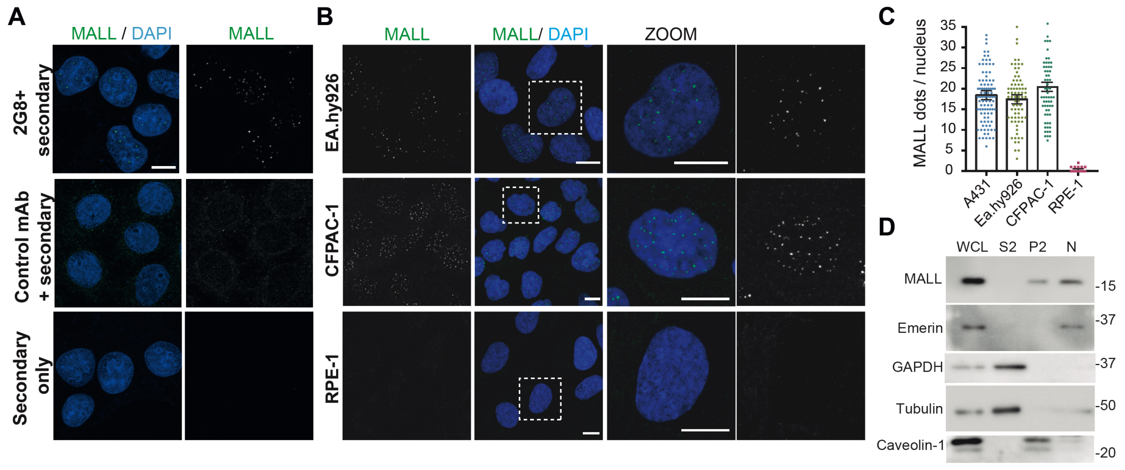

Figure S1. MALL is present in the nucleus in other cell lines. (**A**) A431 cells fixed with cold methanol were stained with mAb 2G8 (upper panel) or with anti Myc 9E10 mAb (control mAb) (middle panel) or omitting the primary antibody (lower panel). (**B**) The indicated cell lines were fixed with cold methanol and were stained with mAb 2G8 and DAPI. (**C**) Quantification of the frequency of nuclear MALL dots per cell in the indicated cell lines (95, 73, 66 and 43 cells of A431, EA.hy926, CFPAC and RPE-1 cell cultures, respectively, were analyzed). Each scored cell is represented by an individual dot in the graph. The mean ± SEM are shown. (**D**) Subcellular fractionation of A431 cells. The indicated subcellular fractions were immunoblotted for MALL with mAb 2G8, and for the following markers: emerin for the nucleus; GAPDH and tubulin for the cytosol; and caveolin-1 for membranes. WCL, whole cell lysate, S2, cytosolic fraction; P2, membrane fraction, N, nuclear fraction.

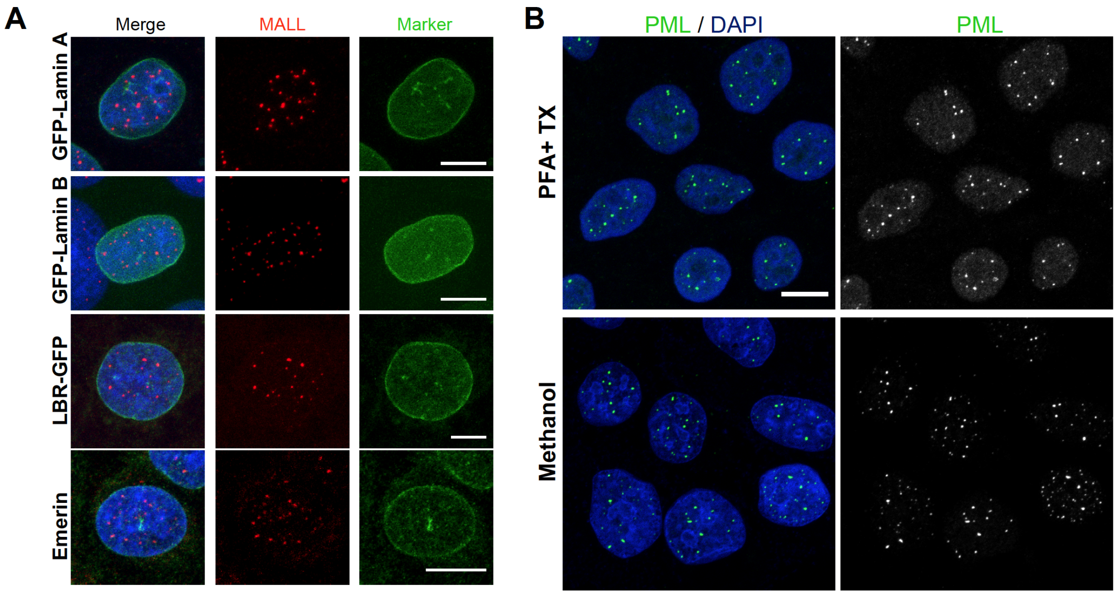

Figure S2. (**A**) MALL is not present in nuclear envelope invaginations. A431cells expressing GFP-lamin A, GFP-lamin B and LBR-GFP were fixed with methanol and stained for endogenous MALL. Control cells were stained for endogenous MALL and emerin (bottom panel). (**B**) A431 cells were fixed and permeabilized with PFA and Triton X-100 (upper panels) or with cold methanol (lower panels) and were stained with anti-PML antibodies. Scale bars, 10 µm.

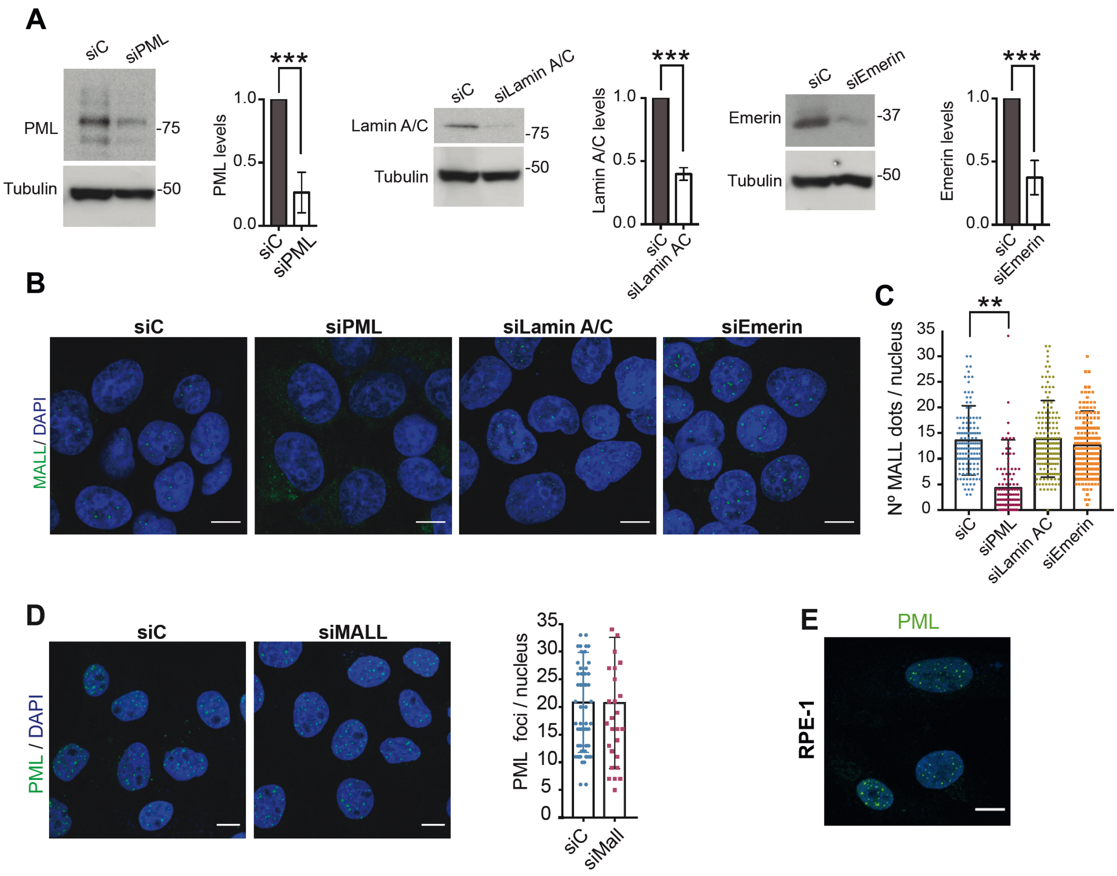

Figure S3. The knockdown of lamin A/C and emerin does not affect the localization of MALL in PML NBs. (**A**) Extracts of A431 transfected with siRNAs targeting PML (siPML), lamin A/C (siLamin A/C), and emerin (siEmerin) for 72 h were immunoblotted for the respective protein and for tubulin, which was used as a loading control. The histograms show the levels of each of the three proteins relative to those in cells transfected with a control siRNA (siC). (**B**) The indicated cells were fixed in methanol and stained for MALL and with DAPI. (**C**) The histogram shows the frequency of nuclear dots of MALL per nucleus in the indicated cells. More than 150 cells per type of cell were analyzed. (**D**) A431 cells transfected with siC or siMALL were stained for PML and with DAPI (left panels). The frequency of PML NBs per nucleus was quantified (right panel). (**E**) RPE-1 cells were stained for endogenous PML. Three independent experiment were performed in (A, C, D) (***, $p<0.001$; (**, $p<0.01$). The mean ± SEM is shown. Scale bars, 10 µm.

A

Organ affected:	Cancer vs. Normal		Cancer vs. Normal	
Bladder cancer				
Brain and CNS cancer	1			
Breast cancer		6		
Cervical cancer		2		
Colorectal cancer		21		1
Esophageal cancer		5		
Gastric cancer				
Head and neck cancer		4		
Kidney cancer	3			
Leukemia				
Liver cancer				
Lung cancer		4		1
Lymphoma				
Melanoma				
Myeloma		1		
Other cancer	3	2		
Ovarian cancer				
**Pancreatic cancer**	3		2	
Prostate cancer		2		
Sarcoma		1		
Significant unique analysis	10	48	2	2
Total unique analysis	341		341	

Threshold (p-value)	$1e^{-3}$	$1e^{-3}$
Threshold (fold change)	2	20
Threshold (gene rank)	10%	10%

10 5 1 % 1 5 10

B

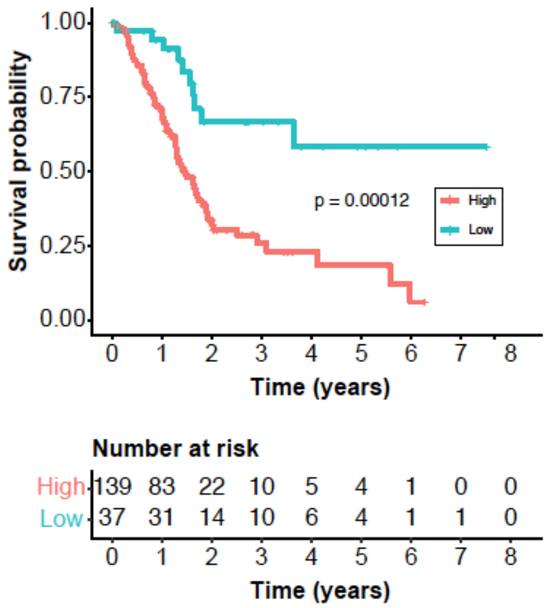

C

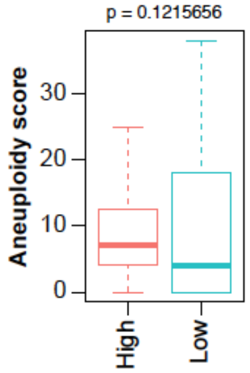

Figure S4. Expression on MALL in cancer. (**A**) Analysis of MALL mRNA expression in cancer versus normal tissue in twenty types of cancer according to Oncomine (accessed on November, 2021). The data were analyzed using two different conditions, as indicated. The red and blue boxes indicate overexpression and downregulation, respectively. The scale color intensity indicates the best rank of the gene in the analyses. The numbers in the boxes show how many analyses were done that fulfilled the specified conditions. Pancreatic cancer is highlighted against a yellow background. (**B**) Correlation between MALL mRNA expression and patient outcome according to the Human Protein Atlas (accessed on November, 2021). (**C**) Aneuploidy score of pancreatic cancer samples expressing high and low levels of MALL mRNA. Wilcox.test was used to calculate the p-value.

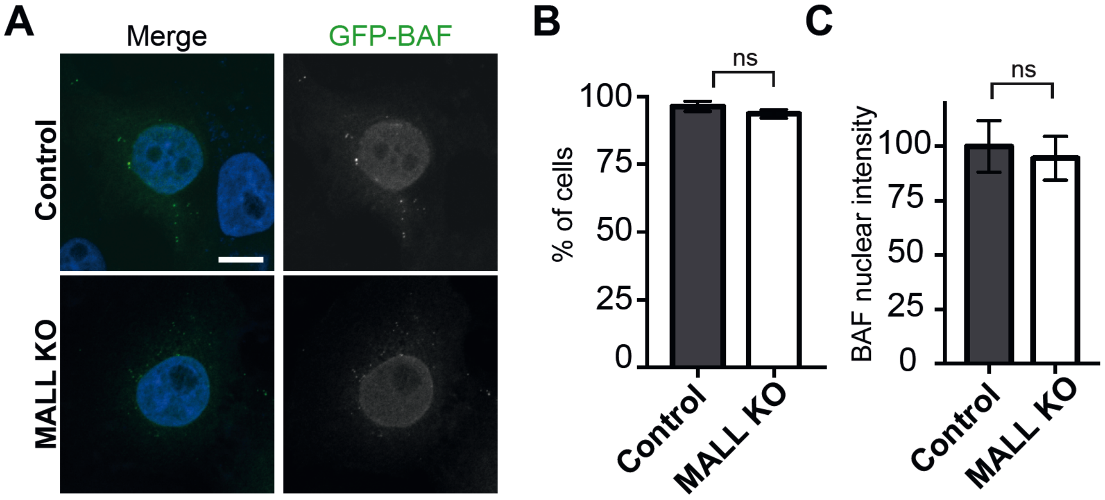

Figure S5. The knockout of MALL does not affect the localization of GFP-BAF. (**A**-**C**) Control or KO MALL cells were transfected with GFP-BAF. After 48 h, cells were fixed with PFA and stained with DAPI (A). The histograms in (B, C) show the percentage of cells with BAF predominantly nuclear and the intensity of the nuclear signal of BAF in control and MALL KO cells, respectively. 122 control and 83 KO MALL cells were analyzed. ns, not significant.
